# Efficacy of heel lifts for insertional Achilles tendinopathy (LIFTIT): A randomised feasibility trial

**DOI:** 10.1002/jfa2.70025

**Published:** 2024-12-19

**Authors:** Jaryd Bourke, Shannon Munteanu, Alessandro Garofolini, Simon Taylor, Peter Malliaras

**Affiliations:** ^1^ Physiotherapy Department School of Primary and Allied Health Care Faculty of Medicine Nursing and Health Science Monash University Melbourne Victoria Australia; ^2^ Discipline of Podiatry School of Allied Health Human Services and Sport La Trobe University Melbourne Victoria Australia; ^3^ Institute for Health and Sport (IHES) Victoria University Melbourne Victoria Australia

**Keywords:** Achilles tendon, heel lift, tendinopathy

## Abstract

**Objectives:**

Insertional Achilles tendinopathy is a common and disabling condition. This trial aimed to determine the feasibility of conducting a parallel group randomised trial to evaluate the efficacy of heel lifts compared to a sham intervention for reducing pain intensity associated with insertional Achilles tendinopathy.

**Methods:**

Twenty‐six people with insertional Achilles tendinopathy were randomised to either the heel lift group or sham intervention group. Outcome measures were obtained at baseline, 4, 8 and 12 weeks. The primary outcome was feasibility, evaluated according to demand (recruitment rate and conversion rate), acceptability, adherence, adverse events and retention. Limited efficacy testing was conducted on secondary outcome measures including pain intensity, function, physical activity, health‐related quality of life, use of co‐interventions and global rating of change.

**Results:**

Between August 25, 2023, and April 7, 2024, we recruited and tested 26 participants (aged 28–65 years, mean [SD] 51 [8]). The pre‐determined thresholds were met for demand, acceptability, adherence, retention, pain intensity, function, quality of life and global rating of change and partly met for adverse events, physical activity and use of co‐interventions. Between 47 and 241, participants will be needed for a fully powered randomised trial.

**Conclusion:**

In its current form, a randomised trial of heel lifts compared to a sham intervention is feasible. However, future triallists may need to consider strategies to manage the risk of adverse events and plan to adjust the analyses to account for the use of co‐interventions.

**Trial registration:** ACTRN12623000721606.

## 1 BACKGROUND

Insertional Achilles tendinopathy (AT) is a common condition, estimated to affect 6% of people in their lifetime [1]. It is characterised by localised tissue pathology and activity‐related pain in the distal 2 cm of the posterior calcaneal insertion [2]. Most people (up to 63%) recover from the condition; however, symptoms can persist for up to 10 years and 24% are unable to return to sport or their previous activity levels [3]. In addition, because of the sensitivity of the posterior heel, many struggle to wear enclosed shoes [4].

The aetiology of insertional AT is multifactorial, often attributed to a sudden increase in load [4]. Other factors that increase tendon load or compromise the integrity of the tendon, such as obesity and corticosteroid injections, may further heighten the risk of tendinopathy [2]. A range of interventions have been proposed for insertional AT, such as calf strengthening exercise, extracorporeal shockwave therapy, orthoses, changing footwear and education about reducing painful activities to a tolerable amount [2, 4]. However, the evidence supporting these interventions is often uncertain or non‐existent [2, 4]. Heel lifts are an alternative intervention that is commonly prescribed for this condition as they are thought to help with symptoms of tendinopathy by reducing compressive strain at the insertion [5, 6].

There is evidence from one recent case‐series study has supported the use of heel lifts for insertional AT, reporting improved pain and gait after 2 weeks of using this intervention [7, 8]. However, there were limitations to this study, including (a) no masking of participants to the allocated intervention and (b) there was an absence of a comparison or no treatment group (e.g. sham). Therefore, it is unclear if the efficacy of the heel lifts was due to specific treatment effects, placebo effects or other factors, such as natural history. Hence, there is a need to conduct a rigorous randomised trial to evaluate the efficacy of heel lifts for insertional AT.

It is essential to assess the feasibility of a large trial given the cost and time involved. Therefore, the primary aim of this study was to evaluate the feasibility of conducting a randomised trial comparing heel lifts versus a sham intervention for reducing pain associated with insertional Achilles tendinopathy. The secondary aims were to provide a signal of efficacy to justify a future main trial and obtain statistical parameters to inform the main trial sample size calculation.

## 2 METHODS

### 2.1 Study design

The efficacy of heel lifts for insertional Achilles tendinopathy (the LIFTIT trial) was a parallel‐group, randomised feasibility trial over 12 weeks. The methods are based on similar trials [9, 10]. Findings are reported according to the CONSORT extension for randomised pilot and feasibility trials (Supplementary File 1) [11], as well as the template for intervention description and replication checklist [12]. There were no deviations from the study protocol. The study was approved by Monash University Human Ethics Committee (39058) and registered in the Australian New Zealand Clinical Trials Registry, ACTRN12623000721606.

### 2.2 Participants

Between August 2023 and April 2024, participants were recruited through Facebook advertisements and emailing people who have previously participated in Monash University studies. People who expressed interest by registering at our trial website or emailing our research team were contacted and screened by a single author (JB). The full eligibility criteria are described in Supplementary File 2; a summary is given below. Participants were included if they were aged between 18 and 65 years old, with clinically and ultrasound diagnosed insertional AT, had pain present for at least 6 weeks and reported a minimum level of pain over the last week of at least a 4 out of 10 on a 11‐point numerical rating scale (10 is the worst pain imaginable). Participants were excluded if they were currently pregnant, had previous Achilles tendon rupture or surgery in the most symptomatic limb(s), had concurrent foot and ankle conditions that were more severe than their worst Achilles tendon pain or had relevant treatment (Achilles injection, orthotics, or heel lifts) in the previous 3 months, prior breast cancer or use of oestrogen inhibitors, taken fluoroquinolone antibiotics in the last 2 years or reported having an inflammatory or neurological medical condition (e.g. rheumatoid arthritis). All assessments were conducted at a single centre (Victoria University, Institute for Health and Sport, Melbourne, Victoria, Australia).

### 2.3 Interventions

**FIGURE 1:**
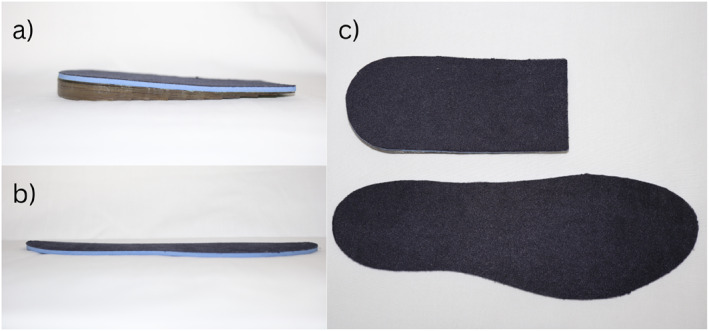
Heel lift (A) and (B) sham intervention. (a) lateral view of heel lift (b) lateral view of sham intervention (c) dorsal view of interventions (top: heel lift and bottom: sham intervention).

Participants in the intervention group were allocated a pair of commercially available (Clearly Adjustable®) 12 mm heel lifts. A 3.2‐mm PPT Ultralux top cover was adhered to the top surface to maximise comfort. The heel lifts were reduced in 1 mm increments if required (e.g. heel slippage) and the final height was recorded at baseline. The decision to use these heel lifts was based on the findings of a previous trial that reported this intervention to be safe and more effective for some clinical outcomes than eccentric calf exercises for mid‐portion AT over 12 weeks [13]. Participants allocated to the control group received a pair of full‐length flat innersoles (hereafter described as the ‘sham intervention’) made of the same materials as the heel lifts (1 mm clear vinyl and 3.2 mm PPT Ultralux). The sham intervention was trimmed to fit the participant's footwear and branded with a ‘Monash Orthotics’ sticker to improve its credibility (Figure 1). Participants were asked to wear their allocated intervention bilaterally for a minimum of 8 h per day. The heel lifts cost approximately $50 per participant and the materials for the sham intervention cost approximately $20 per participant (not accounting for time to assemble and miscellaneous costs such as glue). Both groups also received standardised education on using ‘shoe inserts’ and modifying activities to try and minimise pain to less than 5 out of 10 during activity, as per Silbernagel et al. [14] (Supplementary File 3). Participants were advised to use 500 mg (up to 4 g per day) of paracetamol as needed and were asked to refrain from using other treatments during the 12 weeks they were in the trial.

### 2.4 Outcome measures

#### 2.4.1 Primary outcome: Feasibility

The primary outcome was feasibility, assessed according to demand, acceptability, adherence, adverse events and retention. The measures and thresholds needed to demonstrate feasibility are described below.

#### 2.4.2 Demand

Demand was determined by the recruitment rate (number of participants recruited per month) and the conversion rate (the proportion of participants providing consent of those who met the selection criteria). A recruitment rate of three eligible participants per month and conversion rate of ≥75% were considered acceptable.

#### 2.4.3 Acceptability

Acceptability of the intervention was determined using the credibility/expectancy questionnaire (CEQ) immediately post‐allocation [15]. The intervention was considered acceptable if ≥75% of the intervention group scored ≥50% overall for credibility (questions 1–3) and expectancy (questions 4–6).

#### 2.4.4 Adherence

Adherence was measured at 4, 8 and 12 weeks via a REDCap survey™. Participants were asked to provide information regarding the average number of hours per day and number of days they had worn the heel lifts during the preceding 4 weeks. Adherence was considered acceptable if ≥75% of participants wore their allocated intervention (i.e. heel lift or sham intervention) for an average of ≥5 h per day over the 12 week follow‐up period.

#### 2.4.5 Adverse events

Adverse events from the interventions (such as skin blistering or the occurrence of new pain or injuries in other areas of the foot and body) were assessed at 4, 8 and 12 weeks via a REDCap survey™. Participants were asked to document the type of adverse event and the body location. Two authors (JB, PM) went through the adverse event data to filter out any adverse events that were considered not trial‐related. Serious adverse events were defined as events that are life‐threatening, require hospitilisation or result in persistent or significant difficulty or incapacity. The rate of adverse events was considered acceptable if <15% and none were considered serious.

#### 2.4.6 Retention

Retention rate is the proportion of recruited participants who complete the 12‐week outcome assessment. An ≥80% retention rate in each group was considered acceptable.

#### 2.4.7 Secondary outcomes: Limited efficacy testing

Secondary outcome measures were collected at baseline at Victoria University and 12 weeks (primary endpoint) using a REDCap™ survey, unless stated otherwise. Participants with bilateral symptoms were asked to report for their most painful side (or right side, if the Achilles tendons were equally painful). The selection of efficacy outcome measures for this trial was based on the ICON statement [16].

1. Pain intensity at its worst in the previous week (measured using a 11‐point numerical rating scale (NRS) with terminal descriptors of ‘no pain’ (score = 0) and ‘worst pain possible’ (score = 10)) [17];
2. Pain and disability (measured using the Victorian Institute of Sport Assessment—Achilles (VISA‐A)) [18];
3. Global rating of change scale (measured using a 15‐point Likert scale ranging from a ‘very great deal worse’ to a ‘very great deal better’). This variable was dichotomised with ‘effective’ defined as “somewhat better” or above [19];
4. Function (measured using the lower extremity functional scale (LEFS) [20]);
5. Health‐related quality of life (measured using the VAS component of the EuroQol 5 Dimension 5 Level (EQ‐5D‐5 L) questionnaire [21]);
6. Level of physical activity (measured using the International Physical Activity Questionnaire ‐ short form (IPAQ‐SF) [22]).
7. Number of participants using co‐interventions to relieve their Achilles tendon pain was documented every 4 weeks via a REDCap™ survey.

The feasibility threshold for secondary outcomes was a signal of efficacy for each continuously scored outcome measure, as evidenced by at least a small effect size (Cohen's *d* ≥0.20), less than 20% use of co‐interventions and a greater than 25% difference in the proportion of participants reporting at least ‘somewhat better’ on the perception of the overall treatment effect compared to the control group.

### 2.5 Sample size

As recommended by Julious [23], we aimed to recruit 26 participants (24 plus 2 to allow for a 10% dropout).

### 2.6 Randomisation and blinding

Participants were randomised (1:1 ratio with random permuted block sizes) using the third‐party website Sealed Envelope (https://www.sealedenvelope.com/) to ensure allocation concealment. Participants were blinded via limited disclosure as they were told that the study was comparing two types of ‘shoe inserts’. Primary and secondary outcomes were self‐reported; thus, this trial was assessor blinded. The trial investigator (JB) analysing the data was blinded to group allocation (masking used). However, the clinician (JB) administering the interventions was unable to be blinded due to the nature of the interventions.

### 2.7 Statistical analysis

SPSS® 29 was used for statistical analysis. Data was entered by a single author (JB) and a random sample of 10% was double checked by a second author (SM) for accuracy. Descriptive statistics were used to report the primary feasibility outcomes. Continuous outcomes were explored for normality and differences between groups were compared using mean scores and adjusted mean differences (using analysis of covariance) with Cohen's *d.* Differences in dichotomous‐scaled outcome measures were compared using relative risk, absolute risk and number needed to treat or harm. The standard deviation for the heel lift group at 12 weeks for the pain intensity at its worst and VISA‐A outcome measures were used to calculate sample size estimates for any future randomised trial. To complement point estimates, standard deviations, 95% confidence intervals and *p*‐values were calculated where appropriate.

## 3 RESULTS

**FIGURE 2:**
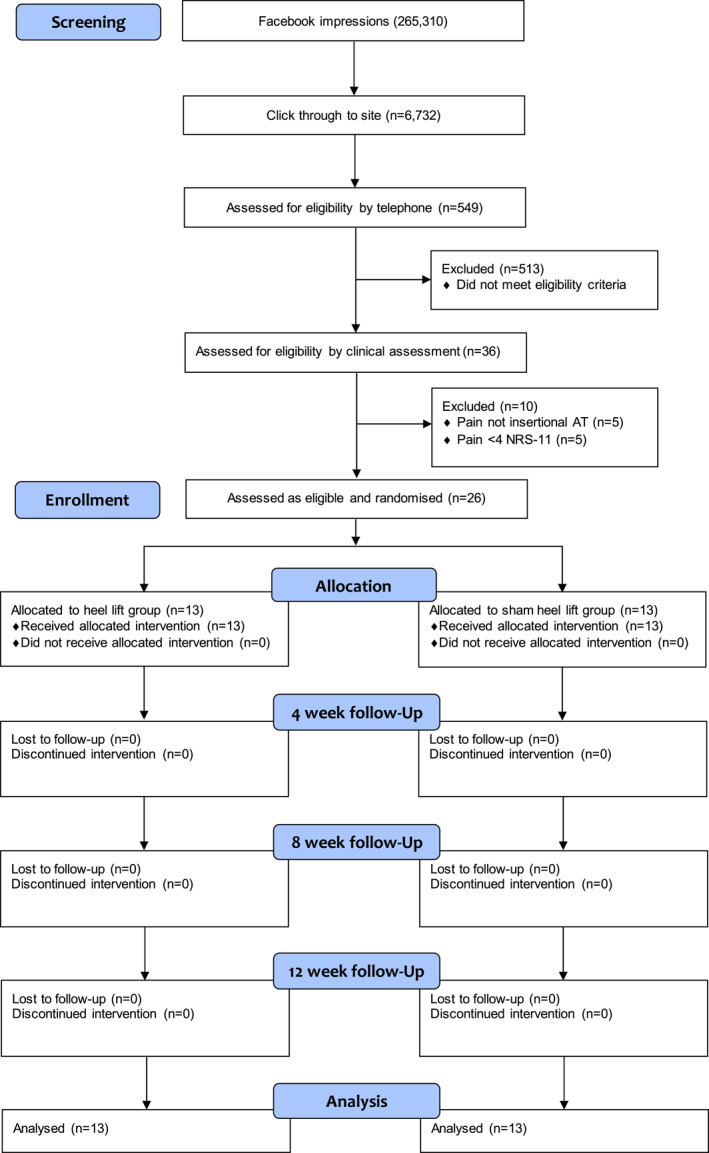
Flow of study.

Five hundred and forty‐nine people were screened by telephone and 26 participants were recruited and randomised (17 men and 9 women, mean age 51.4 ± 8.1). Thirteen participants were allocated to the heel lift group and 13 participants were allocated to the sham intervention group (Figure 2). Both groups were comparable in baseline characteristics (Table 1). Half of the (*n* = 6) participants in the intervention group requested to reduce the height of the heel lift (mean height 10.8 mm).

**TABLE 1:** Participant (*n* = 26) characteristics at baseline.

	Heel lift group (*n* = 13)	Sham (*n* = 13)
Demographics and anthropometrics		
Age (years)	50.8 ± 9.7	51.9 ± 6.4
Sex and gender (female)	5 (39%)	4 (31%)
Height (cm)	168.4 ± 10.8	170.9 ± 7.7
Weight (kg)	91.5 ± 17.4	102.5 ± 28.9
BMI	32.4 ± 6.4	34.8 ± 8.3
Foot posture index score	2.4 ± 2.7	2.7 ± 4.4
Ankle joint dorsiflexion range of motion (degrees)	24.5 ± 8.2	23.7 ± 4.5
Smoking status (yes)	0 (0%)	2 (15%)
Achilles tendinopathy characteristics		
Painful Achilles side (left/right/bilateral)	6(46%)/4(31%)/3(23%)	3 (23%)/4(31%)/6(46%)
Duration of symptoms (months)—median (IQR)	12 (8.5–28)	12 (5.5–24)
Ultrasound imaging (tendon thickening/hypoechoic regions/both)	6(46%)/1(8%)/6(46%)	3(23%)/0(0%)/10(77%)
Footwear		
Women's shoe size (US)	7.8 ± 1.2	8.8 ± 1.4
Men's shoe size (US)	10.6 ± 1.1	10.3 ± 1.7
Pitch (mm)—median (IQR)	10 (9.5–12)	12 (8–13.5)
Main shoes worn (runner)	6 (46%)	9 (69%)
Ethnicity		
United Kingdom	7 (54%)	4 (31%)
Oceanian	2 (15%)	1 (8%)
Southern and eastern Europe	1 (8%)	3 (23%)
North West European	2 (15%)	2 (15%)
Southeast Asian	0 (0%)	1 (8%)
Southern and central Asian	0 (0%)	1 (8%)
North African and middle eastern	1 (8%)	0 (0%)
Unknown/Not reported	0 (0%)	1 (8%)
Self‐reported education		
High school (or equivalent completed)	0 (0%)	3 (23%)
Vocational training	1 (8%)	2 (15.5%)
College/university completed	9 (69%)	3 (23%)
Postgraduate degree completed	3 (23%)	5 (39%)
Self‐reported employment		
Full‐time	12 (93%)	12 (93%)
Part‐time	0 (0%)	1 (8%)
Casual	1 (8%)	0 (0%)
Self‐reported medications^a^		
Anti‐hypertensive	4 (31%)	3 (23%)
Cholesterol	1 (8%)	1 (8%)
Mental health	0 (0%)	2 (15%)
Vitamins and supplements	4 (31%)	1 (8%)
Thyroid	1 (8%)	1 (8%)
Diabetes	1 (8%)	0 (0%)
Other	2 (15%)	2 (15%)
No medications	5 (39%)	7 (54%)
International physical activity questionnaire‐short form		
*Vigorous exercise*		
Yes	9 (69%)	8 (62%)
Days per week	3.3 ± 2.0	2.1 ± 0.9
Minutes per day	81.7 ± 106.7	71.3 ± 37.2
*Moderate exercise*		
Yes	9 (69%)	9 (69%)
Days per week	2.9 ± 1.8	2.1 ± 0.7
Minutes per day	81.0 ± 71.4	99.4 ± 91.7
*Walking*		
Yes	13 (100%)	13 (100%)
Days per week	6.1 ± 1.7	5.8 ± 1.9
Minutes per day	68.1 ± 63.0	71.9 ± 124.3
*Sitting*		
Hours per day	6.4 ± 2.6	8.1 ± 3.6

*Note*: Values are mean ± SD unless otherwise noted.

^a^ Some participants took more than one type of medication.

### 3.1 Primary outcomes

Table 2 provides a summary of the results for the feasibility outcome measures. All thresholds for feasibility besides adverse events were met. Adverse events were common (65%) but not serious, so were considered partly met (Supplementary File 4).

**TABLE 2:** Summary of feasibility outcome measures and thresholds.

Feasibility construct	Outcome	Pre‐determined threshold	Result	Achieved?
Demand	Recruitment rate	3 participants per month	5.2	Yes
	Conversion rate	≥75%	100%	Yes
Acceptability	CEQ	≥75% scored ≥50% for credibility and expectancy	92%	Yes
Adherence	REDCap survey every 4 weeks	≥75% used their intervention ≥5 h per day	92%	Yes
Adverse events	REDCap survey every 4 weeks	<15% and none are considered serious	65%	Partially^a^
Retention	Proportion of participants followed up at 12 weeks	≥80%	100%	Yes

^a^ Although 65% reported an adverse event, none were considered serious.

Abbreviations: CEQ, Credibility/Expectancy Questionnaire.

Four people declined to attend a clinical assessment. The conversion rate of eligible participants was 100% and the recruitment rate ranged from 4 to 6 per month over 5 active months (i.e. we stopped recruiting for 4 months intermittently for Christmas and holidays). Twenty‐five (96%) participants were recruited via Facebook (total spend $2527 AUD; average spend of $97 AUD per person recruited) and the remaining person via emailing people who have previously participated in Monash University studies. All participants completed the study and used their allocated intervention for an average of 6.3 days per week and 9.2 h per day (Supplementary File 5).

Participants in both groups considered their interventions to be credible and they expected to benefit from them (Supplementary file 6). Treatment credibility and expectancy were similar between the heel lift (credibility: 21.8 ± 1.7; expectancy: 18.3 ± 3.1) and sham intervention group (credibility: 22.1 ± 4.6; expectancy: 17.8 ± 6.1).

### 3.2 Secondary outcomes

Table 3 provides a summary of the limited efficacy outcome measures and pre‐determined thresholds. The thresholds for pain intensity at its worst in the previous week, VISA‐A, LEFS, GRoC and QoL outcome measures were achieved. The average use of co‐interventions across the 12 weeks was 15% in the heel lift group and 31% in the sham intervention group, partially meeting the criteria (Supplementary File 7). Physical activity levels were also considered partially met, as all aspects exceeded Cohen's *d* of 0.20, except for moderate exercise minutes (Supplementary File 8).

**TABLE 3:** Summary of the limited efficacy testing outcome measures.

Efficacy measure	Pre‐determined threshold	Result	Achieved?
Pain at its worst (NRS‐11)	Small effect size (*d* ≥0.20)	0.94	Yes
VISA‐A	Small effect size (*d* ≥0.20)	0.87	Yes
LEFS	Small effect size (*d* ≥0.20)	0.79	Yes
QoL	Small effect size (*d* ≥0.20)	0.61	Yes
IPAQ‐SF	Small effect size (*d* ≥0.20)	0.13–0.80	Partially^a^
Use of co‐interventions	<20%	23%	Partially^b^
GRoC	≥25% difference between groups in proportion reporting at least “somewhat better”	31%	Yes

Abbreviations: GRoC, Global Rating of ChangeNRS; IPAQ‐SF, International Physical Activity Questionnaire‐Short Form; LEFS, Lower Extremity Functional Scale; Numerical Rating Scale; QoL, Quality of Life; VISA‐A, Victorian Institute of Sport Assessment—Achilles.

^a^ Minutes doing moderate exercise was Cohen's *d* 0.13 and the rest was ≥0.20.

^b^ Use of co‐interventions was 15% in the heel lift group.

Both groups displayed improvement in self‐reported outcome measures during the study (Table 4). The global rating of change scale showed a 31% difference (*p‐*value 0.046) between groups, significantly in favour of the heel lifts (Supplementary File 9). The pain at its worst in the previous week decreased by an average of 3.2 points in the heel lift group and 2.7 points in the sham intervention group, trending towards there being a significant difference in favour of the heel lifts (adjusted mean difference −1.5, 95% CI ‐3.0 to 0.1, *p* value 0.06). No other significant differences were observed.

**TABLE 4:** Secondary outcome measures at baseline and follow‐up.

Outcome measure	Heel lift	Sham	Adjusted mean difference (95% CI)	*p*‐value	Cohen's *d*
Pain at its worst (NRS‐11)					
Baseline	6.1 ± 1.3	6.8 ± 1.6			
12 weeks	2.9 ± 1.8	4.7 ± 2.0	−1.5 (−3.0 to 0.1)	0.06	0.94
VISA‐A					
Baseline	48.1 ± 14.9	34.6 ± 10.8			
12 weeks	73.3 ± 15.3	59.3 ± 16.8	5.5 (−7.5 to 18.6)	0.39	0.87
LEFS					
Baseline	54.9 ± 7.2	47.5 ± 14.9			
12 weeks	69.1 ± 6.5	60.9 ± 13.0	4.6 (−3.0 to 12.1)	0.22	0.79
EQ‐5D‐5 L VAS					
Baseline	71.8 ± 15.6	64.2 ± 16.9			
12 weeks	72.2 ± 16.1	61.9 ± 17.4	5.4 (−5.9 to 16.6)	0.33	0.61

*Note*: *Statistically significant.

Abbreviations: EQ‐5D‐5 L VAS, EuroQol 5 Dimension 5 Level Visual Analogue Scale; LEFS, Lower Extremity Functional Scale; NRS, Numerical Rating Scale; VISA‐A, Victorian Institute of Sport Assessment—Achilles.

### 3.3 Sample size for main trial

The sample size estimates using the pain at its worst and VISA‐A outcome measures are shown in Supplementary File 10. Using G*Power [24] and an alpha of 5%, we estimate that a fully powered parallel‐group superiority trial would require between 47 and 180 participants at 80% power and 62 to 241 participants at 90% power (assuming there is no attrition).

## 4 DISCUSSION

The primary aim of this study was to evaluate the feasibility of conducting a randomised trial comparing heel lifts versus a sham intervention for reducing pain associated with insertional AT. We found that the pre‐determined feasibility thresholds were met (83%) or partially met (17%) for all criteria, confirming a trial using these methods to be feasible. The secondary aims were to provide a signal of efficacy to justify a future main trial and obtain statistical parameters to inform the main trial sample size calculation. We estimate that between 47 and 241, participants will be needed to detect a moderate to large difference between groups. Our preliminary findings signalled heel lifts may improve pain, function, physical activity, health‐related quality of life and global rating of change. However, it is important to note that this was a feasibility trial, so it is not adequately powered to evaluate efficacy.

Despite establishing feasibility, it should be highlighted that 17 participants reported at least one adverse event, though these were all minor and transient. Not surprisingly, participants in the heel lift group (85%) experienced more adverse events compared to the sham intervention group (46%) with the most common being new foot pain. Developing new areas of pain is common during the initial wear‐in period with heel lifts and has been reported in previous studies using this intervention for mid‐portion AT [13]. Our study gave the participants an investigator's phone number to contact if an adverse event occurred; however, to further mitigate this risk in future trials, we recommend implementing a gradual wearing‐in period. This could involve increasing wear time by 1 hour each day, a practice commonly used with foot orthoses [25], to help participants adjust more comfortably to the heel lifts. We also noted a disparity in the use of co‐interventions between groups. Participants were twice as likely to use an additional treatment in the sham intervention group, despite screening for this in our eligibility criteria. Paracetamol accounted for 53% of the co‐interventions in the sham group, which was permitted in this study. Regardless, this is a covariate that could bias the results, and we recommend future trials consider adding sensitivity analyses to adjust for the use of co‐interventions as needed.

### 4.1 Limitations

The findings of this study need to be interpreted in the context of its strengths and limitations. The study design had several key strengths, including randomisation, concealed allocation and blinded analysis, and the reporting adheres to the CONSORT 2010 statement extension to randomised pilot and feasibility trials [11]. In addition, we have chosen heel lifts that are commercially available and therapeutic efficacy supported by the literature for mid‐portion AT [13]. However, there are limitations we need to acknowledge. First, we had high conversion and retention, which could be attributed to the trial being low demand and single centred or because only one investigator screened, randomised and collected data [26]. However, these findings may be unrealistic for a fully powered trial, and it may be more reasonable to assume a 76% conversion and 80% retention rate as was found in similar trials conducted in Australia recently [13, 27]. Second, this trial used an explanatory design [28]; however, it does not contain any measures to investigate the mechanism (i.e. reducing strain at the insertion [5, 6]), which would be a valuable addition for any future trials.

## 5 CONCLUSIONS

The results of this study suggest that a randomised trial comparing heel lifts to a sham intervention is feasible and relatively safe for people with insertional Achilles tendinopathy. Any future trial will provide much needed evidence regarding this common non‐surgical intervention.

## AUTHOR CONTRIBUTION

**Jaryd Bourke:** Conceptualisation; Investigation; Methodology; Formal Analysis; Writing ‐ Original Draft; Writing ‐ Review and Editing. **Shannon Munteanu:** Conceptualisation; Methodology; Supervision; Formal analysis; Writing ‐ Review and Editing. **Alessandro Garofolini:** Conceptualisation; Supervision; Writing ‐ Review and Editing. **Simon Taylor:** Conceptualisation; Supervision; Writing ‐ Review and Editing. **Peter Malliaras:** Conceptualisation; Methodology; Supervision; Formal analysis; Writing ‐ Review and Editing.

## CONFLICT OF INTEREST STATEMENT

The authors declare no conflicts of interest.

## ETHICS STATEMENT

Ethical approval was obtained from the Monash University Human Ethics Committee (39058) with written informed consent obtained from all participants. Ethical standards adhered to the National Health and Medical Research Council (NHMRC) National Statement on Ethical Conduct in Human Research.

## Supporting information

Supporting Information S1

Supporting Information S2

Supporting Information S3

Supporting Information S4

Supporting Information S5

Supporting Information S6

Supporting Information S7

Supporting Information S8

Supporting Information S9

Supporting Information S10

## Data Availability

Data are available on reasonable request.
