# Supplementary material for: Efficacy of heel lifts for insertional Achilles tendinopathy (LIFTIT): A randomised feasibility trial
Source: J Foot Ankle Res. 2024 Dec 19;17(4):e70025. doi: 10.1002/jfa2.70025 (PMC11658913; doi:10.1002/jfa2.70025)
Supplement: Supplementary file 2 — Supporting Information S2 [file JFA2-17-e70025-s001.docx]

**Supplementary File 2:** Eligibility criteria

| Inclusion | Exclusion |
| --- | --- |
| 1. Aged 18 to 65 years; 2. Symptoms of insertional Achilles tendinopathy in one or both lower limb(s) for >6 weeks; 3. Report maximum Achilles tendon pain severity experienced over the past week that is >3 out of 10 (using a numerical pain rating scale); 4. Regularly use footwear that can accommodate at least 10 mm heel lifts. This is defined as using footwear that can accommodate heel lifts for at least eight hours per day [1]; 5. Be literate in English and able to complete the questionnaires used in this trial (e.g., VISA-A questionnaire); 6. Be willing to not receive any treatment on the involved Achilles tendon(s) (other than those allocated in the current study) during the study period; 7. Be willing and able to attend Victoria University (Melbourne, Australia) on one occasion for assessment.   Insertional Achilles tendinopathy was diagnosed as per the clinical guidelines [2] and musculoskeletal ultrasound [3] using the following criteria:   1. Report pain in the Achilles tendon during or after weight-bearing activities including walking, running or jumping/hopping; 2. Pain in the distal 2 cm Achilles tendon insertion (as described by the patient and palpated by the investigator); 3. Gray-scale musculoskeletal ultrasound of the Achilles tendon(s) showing diffuse or local thickening (anterior-posterior) and/or irregular fibre orientation and/or hypoechoic areas within the insertion of the Achilles tendon. Certain features are commonly associated with insertional Achilles tendinopathy; however, may also exist in asymptomatic individuals [4]. Therefore, if participants exhibit the aforementioned sonographic features accompanied by fluid in the retrocalcaneal bursae, focal calcifications, paratenon thickening or calcaneal cortical anomalies (e.g., spurring); they were not excluded [5]. | 1. Currently pregnant; 2. Achilles tendon disorders that are not insertional (e.g., mid-portion Achilles tendinopathy); 3. Previous Achilles tendon rupture or surgery in the symptomatic lower limb; 4. Injury or pathology of the lower limb and/or back or any condition that, in the opinion of the investigators, may interfere with participation in the study (e.g., chronic ankle instability); 5. Concurrent conditions (ankle or other region) that are more severe (pain numerical rating scale) than their worst insertional Achilles tendinopathy pain; 6. Treatment with foot orthoses or heel lifts within the previous three months; 7. Previous breast cancer/and or use of oestrogen inhibitors; 8. Inflammatory arthritis (e.g., psoriatic arthritis); 9. Neurological disorders (e.g., Charcot-Marie-Tooth disease); 10. Taken fluoroquinolones within the previous two years; 11. Any injection (e.g., corticosteroid) into the Achilles tendon or surrounding area in the previous three months; 12. Any medical condition that deems a participant unsuitable, based on the opinion of the investigators (e.g., type I or II diabetes). |

**References**

1. Kulig K, Reischl F, Pomrantz B, Burnfield J, Mais-Requejo S, Thordarson D, Smith R. Nonsurgical management of posterior tibial tendon dysfunction with orthoses and resistive exercise: A randomised controlled trial. Phys Ther. 2009;89:11-2.
2. de Vos R, van der Vlist AC, Zwerver J, et al. Dutch multidisciplinary guideline on Achilles tendinopathy. Br J Sports Med. 2021;55:1125-34.
3. Khan KM, Forster BB, Robinson J, et al. Are ultrasound and magnetic resonance imaging of value in assessment of Achilles tendon disorders? A two year prospective study. Br J Sports Med. 2003;37:149-53.
4. Khan KM, Forster BB, Robinson J, et al. Are ultrasound and magnetic resonance imaging of value in assessment of Achilles tendon disorders? A two year prospective study. Br J Sports Med. 2003;37:149-53.
5. Leung J, Griffith J. Sonography of chronic Achilles tendinopathy: a case-control study. J Clin Ultrasound. 2008;36:27–32.
