# Supplementary material for: Efficacy of heel lifts for insertional Achilles tendinopathy (LIFTIT): A randomised feasibility trial
Source: J Foot Ankle Res. 2024 Dec 19;17(4):e70025. doi: 10.1002/jfa2.70025 (PMC11658913; doi:10.1002/jfa2.70025)
Supplement: Supplementary file 4 — Supporting Information S4 [file JFA2-17-e70025-s006.docx]

**Supplementary file 4:** Self-reported adverse events. Values are n (%) unless otherwise noted.

| Type of adverse event | Heel lift | Sham | Relative risk (95% CI) | *p*-value | ARI (%) | NNT-H (95% CI) |
| --- | --- | --- | --- | --- | --- | --- |
| Foot  Ankle  Leg  Knee  Hip/back  Blister  Other | 6 (46)  3 (23)  1 (8)  3 (23)  2 (15)  4 (31)  0 (0) | 0 (0)  1 (8)  1 (8)  3 (23)  2 (15)  1 (8)  3 (12) | 13.0 (0.8 to 209.4)  3.0 (0.4 to 25.2)  1.0 (0.1 to 14.3)  1.0 (0.2 to 4.1)  1.0 (0.2 to 6.1)  4.0 (0.5 to 31.1)  0.1 (0.0 to 2.5) | 0.07  0.31  1.00  1.00  1.00  0.19  0.18 | +46  +15  0  0  0  +23  -12 | 2 (1 to 5)  7 (2 to 9)  UTC  UTC  UTC  4 (2 to 17)  4 (2 to 575) |
| Total number of participants experiencing at least 1 adverse event | 11 (85) | 6 (46) | 1.8 (1.0 to 3.4) | 0.06 | +39 | 3 (1 to 20) |

*Significant. UTC = unable to calculate. Some participants had >1 adverse event. “Other” adverse events consisted of shoe inserts being uncomfortable (n=3). Abbreviations: ARI, absolute risk increase; NNT-H, number needed to treat to harm.
