# Supplementary material for: Efficacy of heel lifts for insertional Achilles tendinopathy (LIFTIT): A randomised feasibility trial
Source: J Foot Ankle Res. 2024 Dec 19;17(4):e70025. doi: 10.1002/jfa2.70025 (PMC11658913; doi:10.1002/jfa2.70025)
Supplement: Supplementary file 5 — Supporting Information S5 [file JFA2-17-e70025-s009.docx]

**Supplementary File 5:** Self-reported adherence to the interventions. Values are mean ± SD unless otherwise noted.

|  | Days | | *p*-value | Hours | |  |
| --- | --- | --- | --- | --- | --- | --- |
|  | Heel lift | Sham |  | Heel lift | Sham | *p*-value |
| Baseline to 4 weeks | 6.5 ± 1.1 | 6.4 ± 1.2 | 0.74 | 9.1 ± 2.6 | 9.2 ± 1.4 | 0.85 |
| 4 to 8 weeks | 6.2 ± 1.4 | 6.5 ± 1.0 | 0.52 | 9.1 ± 3.1 | 9.1 ± 2.0 | 1.00 |
| 8 to 12 weeks | 6.2 ± 1.5 | 6.2 ± 1.6 | 1.00 | 9.5 ± 4.0 | 9.3 ± 1.8 | 0.90 |

*Statistically significant. The average number of hours (per day) and days (per week) the participants used their allocated shoe inserts in the preceding four weeks.
