# Supplementary material for: Efficacy of heel lifts for insertional Achilles tendinopathy (LIFTIT): A randomised feasibility trial
Source: J Foot Ankle Res. 2024 Dec 19;17(4):e70025. doi: 10.1002/jfa2.70025 (PMC11658913; doi:10.1002/jfa2.70025)
Supplement: Supplementary file 6 — Supporting Information S6 [file JFA2-17-e70025-s003.docx]

**Supplementary file 6**: Credibility/Expectancy Questionnaire overall scores. Values are mean ± SD.

| Question | Heel lift | Sham | *p-*value |
| --- | --- | --- | --- |
| 1. At this point, how logical does the treatment offered to you seem? | 8.5 ± 0.9 | 8.1 ± 1.2 | 0.36 |
| 2. At this point, how successful do you think the treatment will be in reducing your Achilles tendon symptoms? | 6.6 ± 0.8 | 7.0 ± 1.6 | 0.45 |
| 3. How confident would you be in recommending the treatment to a friend who is experiencing similar problems? | 6.8 ± 0.9 | 7.0 ± 2.0 | 0.72 |
| 4. By the end of the treatment period, how much improvement in your Achilles tendon symptoms do you think will occur? | 5.8 ± 1.6 | 5.8 ± 2.0 | 1.0 |
| 5. At this point, how much do you really feel the treatment will help you to reduce your Achilles tendon symptoms? | 6.8 ± 1.0 | 6.4 ± 2.0 | 0.47 |
| 6. By the end of the treatment period, how much improvement in your Achilles tendon symptoms do you really feel will occur? | 5.7 ± 1.7 | 5.6 ± 2.2 | 0.93 |
| Credibility (questions 1 to 3) | 21.8 ± 1.7 | 22.1 ± 4.6 | 0.87 |
| Expectancy (questions 4 to 6) | 18.3 ± 3.1 | 17.8 ± 6.1 | 0.78 |

Mean ± SD. Maximum score for Credibility and Expectancy is 27 points each.
