# Supplementary material for: Efficacy of heel lifts for insertional Achilles tendinopathy (LIFTIT): A randomised feasibility trial
Source: J Foot Ankle Res. 2024 Dec 19;17(4):e70025. doi: 10.1002/jfa2.70025 (PMC11658913; doi:10.1002/jfa2.70025)
Supplement: Supplementary file 7 — Supporting Information S7 [file JFA2-17-e70025-s007.docx]

**Supplementary Figure 7:** Self-reported use of rescue medication and co-interventions. Values are n (%) unless otherwise noted

|  | Heel lift | Sham | Relative risk (95% CI) | *p*-value | Absolute risk reduction (%) |
| --- | --- | --- | --- | --- | --- |
| Baseline to 4 weeks  Paracetamol  Manual therapy  Exercise  Rest  New Shoes  **Total number of participants** | 0 (0)  1 (8)  0 (0)  0 (0)  1 (8)  **2 (15)** | 4 (31)  0 (0)  1 (8)  1 (8)  0 (0)  **5 (39)** | **0.40 (0.09 to 1.70)** | **0.22** | **+24** |
| 4 to 8 weeks  Paracetamol  Manual therapy  Rest  New shoes  **Total number of participants** | 0 (0)  1 (8)  0 (0)  2 (15)  **2 (15)** | 4 (31)  0 (0)  1 (8)  1 (8)  **5 (39)** | **0.40 (0.09 to 1.70)** | **0.22** | **+24** |
| 8 to 12 weeks  Exercise  Paracetamol  Manual therapy  Rest  Orthotics  Ice/heat  **Total number of participants** | 2 (15)  0 (0)  1 (8)  1 (8)  0 (0)  0 (0)  **2 (15)** | 1 (8)  2 (15)  1 (8)  1 (8)  1 (8)  1 (8)  **2 (15)** | **1.00 (0.17 to 6.07)** | **1.00** | **0** |

Number of participants using co-interventions, and the types used in the preceding 4 weeks. *Statistically significant. Some participants used more than 1 co-intervention.
