# Supplementary material for: Efficacy of heel lifts for insertional Achilles tendinopathy (LIFTIT): A randomised feasibility trial
Source: J Foot Ankle Res. 2024 Dec 19;17(4):e70025. doi: 10.1002/jfa2.70025 (PMC11658913; doi:10.1002/jfa2.70025)
Supplement: Supplementary file 8 — Supporting Information S8 [file JFA2-17-e70025-s008.docx]

**Supplementary File 8:** IPAQ-SF at 12 weeks. Values are mean ± SD unless otherwise noted.

| **Type of activity** | Heel lift | Sham | Adjusted mean difference (95% CI) | *p*-value | Cohen’s *d* |
| --- | --- | --- | --- | --- | --- |
| **Vigorous exercise**  Yes (%)  Days per week  Minutes per day  **Moderate exercise**  Yes (%)  Days per week  Minutes per day  **Walking**  Yes (%)  Days per week  Minutes per day  **Sitting**  Hours per day | 10 (77)  3.4 ± 1.9  53.3 ± 35.6  9 (69)  4.7 ± 2.1  104.0 ± 98.3  11 (85)  6.1 ± 1.6  82.5 ± 65.4  4.5 ± 1.4 | 9 (69)  2.9 ± 1.7  79.3 ± 59.2  9 (69)  3.4 ± 2.1  116.3 ± 95.8  13 (100)  6.4 ± 1.1  61.3 ± 50.5  6.0 ± 2.2 | 0.1 (-1.8 to 2.1)  -35.6 (-81.9 to 10.7)  0.6 (-01.7 to 2.8)  21.6 (-100.1 to 142.2)  -0.4 (-1.7 to 0.9)  22.2 (-24.2 to 68.7)  -1.3 (-2.8 to 0.3) | 0.89  0.12  0.58  0.70  0.50  0.33  0.10 | 0.28  0.57  0.63  0.13  0.25  0.36  0.80 |

*Statistically significant. Vigorous physical activities refer to activities that take hard physical effort and make you breathe much harder than normal. days. Moderate activities refer to activities that take moderate physical effort and make you breathe somewhat harder than normal. Abbreviations: IPAQ-SF, International Physical Activity Questionnaire-Short Form.
