# Supplementary material for: Efficacy of heel lifts for insertional Achilles tendinopathy (LIFTIT): A randomised feasibility trial
Source: J Foot Ankle Res. 2024 Dec 19;17(4):e70025. doi: 10.1002/jfa2.70025 (PMC11658913; doi:10.1002/jfa2.70025)
Supplement: Supplementary file 9 — Supporting Information S9 [file JFA2-17-e70025-s002.docx]

**Supplementary File 9:** Global Rating of Change (GroC) scale at 12 weeks.

|  | Heel lift | Sham | Relative risk (95% CI) | *p*-value | ABI (%) | NNT-B (95% CI) |
| --- | --- | --- | --- | --- | --- | --- |
| GroC | 13 (100%) | 9 (69%) | 1.44 (1.01 to 2.08) | 0.046* | +31 | 3 (2 to 18) |

*Statistically significant. Abbreviations: ABI, Absolute benefit increase. NNT-B, Number needed to treat to benefit. The heel lifts group was considered the experimental group for all calculations. Participants rated improvement on a 15-point Likert scale of perception of treatment effectiveness, which was dichotomised according to ‘treatment effectiveness’, where ‘treatment effectiveness’ was defined as ‘somewhat better’ or above, which were the five best scores on the scale.
