# Supplementary material for: Efficacy of heel lifts for insertional Achilles tendinopathy (LIFTIT): A randomised feasibility trial
Source: J Foot Ankle Res. 2024 Dec 19;17(4):e70025. doi: 10.1002/jfa2.70025 (PMC11658913; doi:10.1002/jfa2.70025)
Supplement: Supplementary file 10 — Supporting Information S10 [file JFA2-17-e70025-s004.docx]

| Outcome measure | MID | SD | Cohen’s *d* | Cohen’s *f* | Estimated sample size at 80% power | Estimated sample size at 90% power |
| --- | --- | --- | --- | --- | --- | --- |
| Pain at its worst (NRS-11)  VISA-A | 1.5 points^1^  6.5 points^2^ | 1.8  15.3 | 0.83  0.42 | 0.42  0.21 | 47  180 | 62  241 |

**Supplementary File 10**: Sample size estimates

Using an f-test, analysis of covariance (ANCOVA) in G* power. Abbreviations: MID, minimal important difference; SD, standard deviation from the intervention group.

1. Salaffi F, Stancati A, Silvestri C, Ciapetti A, Grassi W. Minimal clinically important changes in chronic musculoskeletal pain intensity measured on a numerical rating scale. Eur J Pain. 2004;8:283-91. doi:10.1016/j.ejpain.2003.09.004.
2. McCormack J, Underwood F, Slaven E, Cappaert T. The minimum clinically important difference on the VISA-A and LEFS for patients with insertional Achilles tendinopathy. Int J Sports Phys Ther 2015;10(5):639-44.
